# Expression of TSLP and Downstream Molecules IL-4, IL-5, and IL-13 on the Eye Surface of Patients with Various Types of Allergic Conjunctivitis

**DOI:** 10.1155/2016/5072781

**Published:** 2016-07-18

**Authors:** Xiaofen Zheng, Juan Yao, Bing Li

**Affiliations:** Department of Corneal Diseases, Shanxi Eye Hospital, Fu Dong Street No. 100, Xinghualing District, Taiyuan City, Shanxi Province, China

## Abstract

*Background.* The pathogenesis of allergic conjunctivitis has not been clearly established. Moreover, previous studies fail to consider human models of allergic conjunctivitis. This study investigated the expression of thymic stromal lymphopoiet in TSLP and its downstream molecules in conjunctival scrappings and tear.* Methods.* This cross-sectional study compares patients with vernal keratoconjunctivitis (VKC), seasonal allergic conjunctivitis (SAC), and perennial allergic conjunctivitis (PAC) with normal controls. There are 80 people recorded in Shanxi Eye Hospital. Increasingly, 20 are with VKC, 20 are with SAC, 20 are with PAC, and the remaining 20 are normal controls. Conjunctiva were harvested for total RNA extraction and gene expression by real-time polymerase chain reaction. Epithelial cells were collected to make pathological sections for immunohistochemical staining. Human tears were evaluated by Luminex microbead assay. A *P* value less than 0.05 from Dunnett's post hoc test in SPSS means a statistical significant distinction.* Results.* Positive expression in conjunctival cells of patients with allergic conjunctivitis. The expression of TSLP and IL-4, IL-5, and IL-13 mRNA shows a statistically significant difference (*P* < 0.05). TSLP and IL-4, IL-5, and IL-13 concentrations show a statistically significant difference (*P* < 0.01).* Conclusions.* This study suggests that TSLP and downstream molecules are expressed in patients with various types of allergic conjunctivitis.

## 1. Introduction

Allergic conjunctivitis is one of the most common ocular surface diseases. In recent years, the incidence of allergic conjunctivitis showed an increasing trend with the damage of natural environment, increase in the regional haze, frequent use of poor-quality cosmetics, wearing contact lenses, and other factors [1]. The disease ranges in severity from mild forms, such as seasonal allergic conjunctivitis (SAC) and perennial allergic conjunctivitis (PAC) that can still interfere significantly with the quality of life, to severe cases, such as vernal keratoconjunctivitis (VKC) [2] and atopic keratoconjunctivitis [3] that may be complicated by corneal damage and may have the potential to cause permanent vision loss. Allergic conjunctivitis pathogenesis is very complex, but the mechanism is still not very clear. Little was known about the regulation of the development of Th2 type allergic inflammation locally at mucosal surfaces until studies identified a novel proallergic molecule, thymic stromal lymphopoietin (TSLP), a kind of interleukin 7-like cytokine, which can strongly activate dendritic cells through interaction with TSLP receptor (TSLPR) expressed by dendritic cells [4, 5], and then TSLP-treated dendritic cells express OX40 ligands (OX40L) that interact with OX40 to prime CD4+ T cells to produce proallergic cytokines IL-4, IL-13, and IL-5 to induce an inflammatory Th2 type response and initiate allergic inflammation [6–9]. In recent years, short ragweed pollen-triggered allergic conjunctivitis mouse models were excited and MyD88 knockout (MyD88−/−) ones were established. In the animal tests, scholars [10–13] confirmed that TLR4 was produced by conjunctival epithelium through TLR4-dependent innate immune pathways and then stimulated Th2 cell-mediated allergic inflammation of the ocular surface through TSLP-TSLPR and OX40L-OX40 signaling pathways. In regard to basic research aspects, although there are related studies using human corneal epithelial cells [13], they can only simulate the pathological process of similar state close to the human body. For the human experiment of different severity, different stages of acute or chronic condition, and different types of patients with allergic conjunctivitis, further exploration is also needed. The present study sought to investigate the expression of TSLP and its downstream molecules IL-4, IL-13, and IL-5 in the conjunctival tissue of patients with allergic conjunctivitis and tear.

## 2. Material and Methods

### 2.1. Inclusion Criteria [14]

(*1) Vernal Keratoconjunctivitis (VKC)*. It typically appears in boys between the ages of 4 and 12 years and disappears after puberty. Intense itching, tearing, and photophobia are the typical symptoms of VKC. Disease exacerbation can be triggered either by allergen reexposure or, more frequently, by nonspecific stimuli such as sunlight, wind, and dust. The tarsal form of VKC is characterized by irregularly sized hypertrophic papillae, leading to a cobblestone appearance of the upper tarsal plate. The limbal form Horner-Trantas dots and papillae are deposited at the limbus, which may appear thickened and opacified for 360°. Punctate keratitis, epithelial macroerosions, ulcers, and plaques are signs of corneal involvement and resolve with different levels of scarring. 

(*2) SAC.* It is more prevalent from the spring to fall seasons when pollen levels are high. The hallmark symptom is intermittent itching; however, tearing, conjunctival redness, eyelid swelling, and small papillary hypertrophy of tarsal conjunctiva are common but are nonspecific signs. Signs and symptoms arise and subside depending on the patient's exposure to the offending allergen(s). An accurate medical history followed by allergy testing may identify the specific sensitization.

(*3) PAC. *It is present all year round due to allergens, such as dust mites, animal dander, and molds, or due to multiple sensitization instances. It is characterized by the same signs and symptoms as SAC; however, PAC is a chronic condition, with persistent, frequently mild symptoms, enhanced by higher or longer exposure to allergens and exacerbated by nonspecific irritating factors.

### 2.2. The Exclusion Criteria [15]

The exclusion criteria are as follows:

1. Exclusion of people with a history of other keratoconjunctivitis in addition to allergic conjunctivitis.
2. Eczema, atopic dermatitis, urticaria, systemic lupus erythematosus, rheumatoid arthritis, and other systemic allergic case histories.
3. Tear and lacrimal duct diseases.
4. Eye surgery and medication history.
5. The history of wearing contact lens.

The cross-sectional study was conducted on all patients presenting to the clinic at the Shanxi Eye Hospital in China from May 2013 to August 2014. A total of 20 patients (40 eyes) were selected with vernal keratoconjunctivitis (VKC), as well as 20 patients (40 eyes) with SAC, 20 patients (40 eyes) with PAC, and 20 normal controls (40 eyes; these patients had no eye disease). The study was conducted in accordance with the provisions of the Declaration of Helsinki for research involving human subjects. The Ethical Review Committee of the Shanxi Ophthalmic Center approved the study (approval ID: 2013064), and written informed consent was obtained from all study participants.

### 2.3. Immunohistochemical Staining

After completing the examination of patients with allergic conjunctivitis and complex standard normal population, conjunctival scrapings were collected from conjunctival epithelial cells using immunohistochemical methods. Proparacaine hydrochloride eye drops (Alcon, Puurs, BE) were instilled in the ocular surface of the patients' eyes 1-2 times at intervals of 3 minutes. After the materials were scraped, they were immediately applied to the slides, naturally dried for 10 minutes, and then placed in 95% alcohol for 20 minutes to immersion fixation. Cell cultures were permeabilized with 0.2% Triton X-100 in phosphate-buffered saline at room temperature for 10 minutes. Indirect immunostaining was performed according to previously published methods [16–18] with primary rabbit antibodies against human TSLP, IL-4, IL-5, and IL-13 (all at 1 : 500; 1 Ug/mL) for the night at 4°C; primary antibody was applied and incubated for 1 hour at room temperature and for 30 minutes at room temperature, then dropping the goat anti-rabbit biotinylated second antibody (Poway, CA) for 30 minutes. The samples were incubated for 5 minutes with diaminobenzidine (DAB) to give a brown or red stain (optimized for each antibody) and were counterstained with Mayer's hematoxylin, as well as 100% and 95% gradient alcohol dehydration two times. The antibody dilution was used as control. Then, each group was examined and photographed with a microscope equipped with a digital camera (Eclipse E400 with a DS-Fi1, Nikon, NY, USA).

### 2.4. Total RNA Extraction, Reverse Transcription, and Quantitative Real-Time Polymerase Chain Reaction

Conjunctival epithelial cells were collected by impression cytology. Proparacaine hydrochloride eye drops (Alcon, Puurs, BE) were used for topical anesthesia. Sterile microforceps gripping disinfection trapezoid nitrocellulose membrane (Shanxi Medical University Laboratories, Taiyuan, CN) were lightly attached to the conjunctiva of the eye in patients with temporal side, top, and bottom for 10 s and then removed. The conjunctiva cells were obtained, and their epithelia were dissolved in the lysis buffer (Buffer RLT, RNeasy Kit, Qiagen, Valencia, CA) without homogenizing to prevent stromal cell contamination. Total RNA was extracted (RNeasy Micro or Mini Kit, Qiagen) according to the manufacturer's instructions, quantified by a spectrophotometer (NanoDrop ND-1000, Thermo Scientific, MA, USA), and stored at –80°C before use. First-strand cDNA was synthesized by M-MuLV reverse transcription with random hexamers (Ready-To-Go You-Prime First-Strand Beads, GE Healthcare, NJ, USA), as previously described.

Real-time polymerase chain reaction (PCR) was performed with specific primers and probes (using TaqMan Gene Expression Assays and TaqMan Gene Expression Master Mix (Applied Biosystems, CA, USA) in a QPCR System (StepOnePlus, Stratagene, CA, USA)). IDs (TaqMan Gene Expression Assay IDs, Applied Biosystems) for human glyceraldehyde-3-phosphate dehydrogenase (GAPDH), TSLP, IL-4, IL-5, and IL-13 were hs99999905_m1, hs00263639_m1, Hs00174122_m1, Hs01548712_g1, and Hs00174379_m1, respectively. Results were analyzed by the comparative threshold cycle method [19, 20] and normalized by GAPDH, as previously reported [21, 22].

### 2.5. Luminex Detection Tear TSLP, IL-4, IL-5, and IL-13 Cytokine Levels

A total of 4 *μ*L of conjunctival scrapings was collected from the patient's eye using a 1 *μ*L capillary tube, which was quickly transferred into the Eppendorf tube containing 6 *μ*L of 1% BSA solution and stored at –80°C. For using in the assay, the sample was thawed at room temperature and was added to the tear sample assay buffer in a 1 : 1 dilution; detection of cytokines was performed in strict accordance with the Luminex kit instructions.

### 2.6. Statistical Analysis

The statistical analysis was performed using SPSS for Windows 17.0 (SPSS Inc.). The relative expression and protein concentration values of TSLP, IL-4, IL-5, and IL-13 in the normal control, VKC, SAC, and PAC groups were normal as confirmed by the Kolmogorov-Smirnov test (*P* > 0.05) (results shown were the mean ± standard deviation [SD]). The one-way analysis of variance test was used to make comparisons among three or more groups, followed by Dunnett's post hoc test. *P* values <0.05 were considered statistically significant.

## 3. Results

### 3.1. Immunohistochemistry of TSLP, IL-4, IL-5, and IL-13

**Figure 1:**
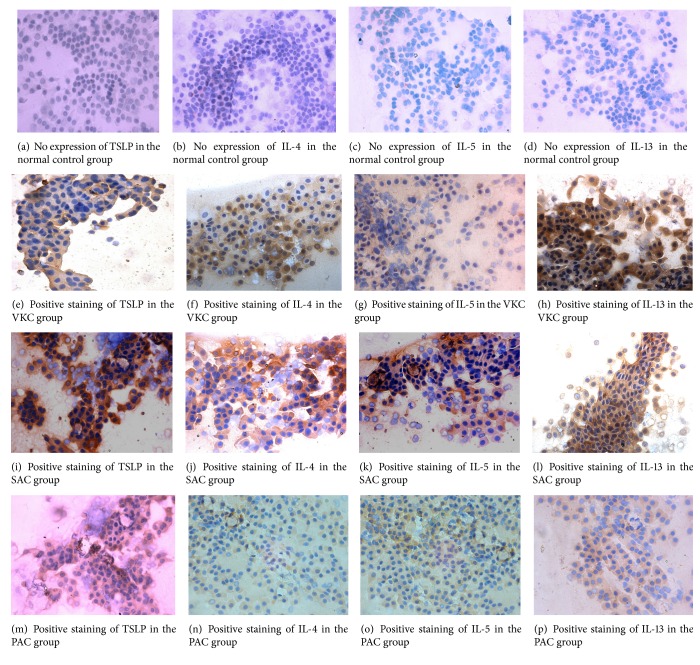
Detection of TSLP, IL-4, IL-5, and IL-13 protein expression by immunohistochemistry (×400). ((a), (b), (c), (d)) No expression of TSLP, IL-4, IL-5, and IL-13 in the normal control group. ((e), (f), (g), (h)) Positive staining of TSLP, IL-4, IL-5, and IL-13 in the VKC group. ((i), (j), (k), (l)) Positive staining of TSLP, IL-4, IL-5, and IL-13 in the SAC group. ((m), (n), (o), (p)) Positive staining of TSLP, IL-4, IL-5, and IL-13 in the PAC group. Brown granules showed positive staining.

Immunohistochemical staining confirmed an increase in TSLP, IL-4, IL-5, and IL-13 production in the eyes with various types of allergic conjunctivitis. As shown in Figure 1, the conjunctival tissues of various types of allergic conjunctivitis displayed stronger TSLP, IL-4, IL-5, and IL-13 staining throughout the entire epithelium than did those of normal controls. These data indicated that TSLP, IL-4, IL-5, and IL-13 protein production by ocular surface epithelia increase in various types of allergic conjunctivitis, suggesting the possible involvement of TSLP, IL-4, IL-5, and IL-13 in allergic development.

### 3.2. mRNA Expression of TSLP, IL-4, IL-5, and IL-13 by Real-Time PCR

**Figure 2:**
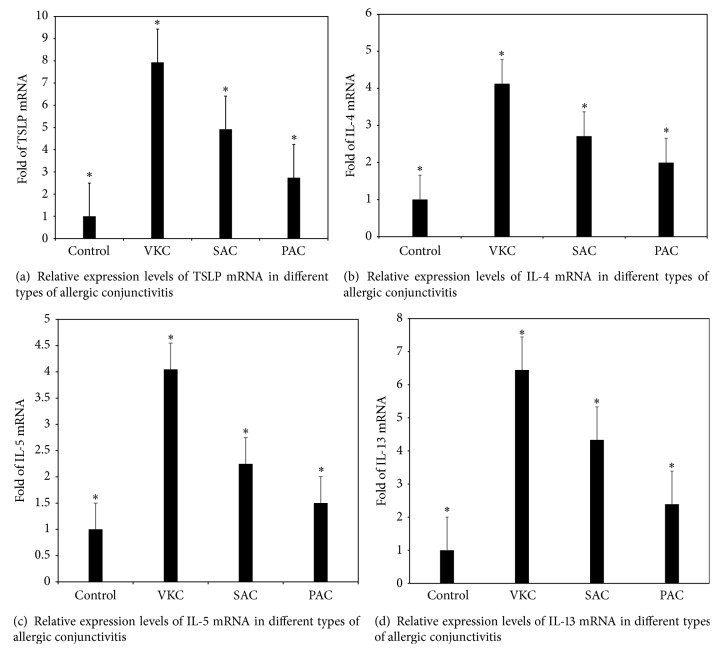
Relative expression levels of TSLP mRNA, IL-4 mRNA, IL-5 mRNA, and IL-13 mRNA in different types of allergic conjunctivitis. TSLP mRNA, IL-4 mRNA, IL-5 mRNA, and IL-13 mRNA expression levels are presented as relative fold in various types of allergic conjunctivitis over the normal controls, which were evaluated by RT and real-time PCR using gene expression assay, with GAPDH as an internal control. Results shown are the mean ± SD. ^*∗*^ *P* < 0.05; each group *n* = 20.

Significant differences were reported in the expression of TSLP, IL-4 mRNA, IL-5 mRNA, and IL-13 mRNA in the four groups of subjects (*F* = 71.67, 51.32, 220.18, and 162.49, *P* < 0.001). Compared with the control group, the VKC, SAC, and PAC groups had an increased expression of the ocular surface TSLP, IL-4 mRNA, IL-5 mRNA, and IL-13 mRNA; the differences were statistically significant (*P* < 0.05); the expression of TSLP, IL-4 mRNA, IL-5 mRNA, and IL-13 mRNA in the VKC group was significantly higher than in the SAC and PAC groups; the difference was statistically significant (*P* < 0.05). The expression of TSLP mRNA, IL-4 mRNA, IL-5 mRNA, and IL-13 mRNA in the SAC group was significantly higher than in the PAC group (*P* < 0.05). These results suggested that the expression of TSLP mRNA, IL-4 mRNA, IL-5 mRNA, and IL-13 mRNA increases in these three groups of allergic conjunctivitis, suggesting the possible involvement of TSLP, IL-4, IL-5, and IL-13 in allergic development (Figure 2).

### 3.3. Comparison of Protein Concentration Results of TSLP, IL-4, IL-5, and IL-13 in Tears

In the samples of the normal control group, the cytokines TSLP, IL-4, IL-5, and IL-13 were not detected; however, these cytokines were detected in all the samples in the VKC, SAC, and PAC groups (Table 1). Test results showed a significant difference in the tear TSLP, IL-4, IL-5, and IL-13 expression levels (*P* < 0.001) among the four groups. The tear TSLP, IL-4, IL-5, and IL-13 expression levels in different types of allergic conjunctivitis were found elevated to varying degrees in the VKC, SAC, and PAC compared with the control group; the differences were statistically significant (*P* < 0.001). TSLP, IL-4, IL-5, and IL-13 concentrations in the VKC group were significantly higher than in the SAC and PAC groups; the difference was statistically significant (*P* < 0.001). These concentrations were significantly higher in the SAC group than in the PAC group; the difference was statistically significant (*P* < 0.001).

## 4. Discussion

Allergic conjunctivitis is mainly mediated by lgE I-type hypersensitivity caused by a very common type of noninfectious ocular inflammatory disease. Its main symptoms are itching, often conjunctival hyperemia, edema, nipple eyelids, follicular hyperplasia, and other signs. It often causes significant eye discomfort, seriously affecting people's daily lives and work.

Classification of different types of allergic conjunctivitis has been controversial (Hannouche and Hoangxuan [23], etc.). According to the clinical manifestations, course, and prognosis differences, allergic conjunctivitis is divided into five types: SAC, PAC, VKC, giant papillary conjunctivitis, and atopic keratoconjunctivitis. Although the majority of allergic diseases are not serious ocular complications and the majority of patients can be clinically diagnosed and treated properly, there are still some patients that fail to be accurately diagnosed on time and hence do not get timely treatment. Long term use of antibiotics and other nontargeted drugs, side effects of drugs, and delayed diagnosis even cause irreversible visual impairment. Therefore, selected clinical trials are more common than the confusing three allergic conjunctivitis types: SAC, PAC, and VKC.

Immunohistochemistry, real-time PCR, and Luminex technology were used to measure the expression of TSLP, IL-4, IL-5, and IL-13 in the tears and conjunctival cell of the normal population and patients with three types of allergic conjunctivitis. Previous studies used enzyme-linked immunosorbent assay or flow cytometry to detect the concentrations of inflammation factors in tears. The Luminex chip technologies [24, 25] are integrated flow cytometry, laser technology, digital signal processing, and traditional chemical technology with the greatest features of high-throughput and high-flexibility. The study approved by Luminex detected TSLP and its downstream molecule concentrations in patients with three types of allergic conjunctivitis, but TSLP was not detected in the normal control group. The expression of four factors was significantly higher in patients with VKC than in patients with SAC and PAC. The expression of four factors was higher in patients with SAC than in patients with PAC. In this study, real-time PCR was used to detect the TaqMan probe method, which was highly specific and sensitive. TaqMan probe also detected that three types of allergic conjunctivitis conjunctival epithelial cells of patients had significantly increased TSLP and its downstream molecules compared with the normal population; the highest expression was observed in the VKC group and the lowest was observed in the PAC group, while the expression of TSLP and its downstream molecules was significantly higher in the VKC group than in the SAC and PAC groups, with the expression in the SAC group being higher than that in the PAC group. These results showed that, in allergic conjunctivitis, TSLP and its downstream molecules, as an important inflammatory factor, not only are involved in the pathogenesis of VKC, SAC, and PAC but also indicated that the expression level of these inflammatory factors was positively correlated with the severity of the disease. We found that TSLP in patients with VKC, whether from the mRNA expression or from protein expression, is significantly higher. Among the three types of conjunctivitis, signs and symptoms of VKC are the most severe ones, being more common in children and teenagers, with recurrent disease and delayed healing. Severe cases can damage the eyesight. The two types of allergic conjunctivitis are often associated with other histories of allergic diseases, such as allergic rhinitis, asthma, and atopic dermatitis. We found that, in patients with SAC and PAC, TSLP and its downstream molecules increased from mRNA levels and protein levels, but their expression was lower than that of VKC, which may be because the two types of allergic conjunctivitis are often associated with other histories of allergic diseases, such as allergic rhinitis, asthma, and atopic dermatitis, but the symptoms and signs of the patients were less than VKC. Our study also found that PAC was lower compared to SAC, which may be due to PAC in patients with mild clinical symptoms; PAC is characterized by mild symptoms, long duration of the disease, long term use of drugs, and other nontargeted drugs. Patients were less sensitive to drugs, so TSLP and downstream molecules IL-4, IL-5, and IL-13 levels were lower than those in SAC patients.

In short, although in the past animal models of allergic conjunctivitis were used, normal corneal epithelial cell culture studies on the role and mechanisms of TSLP in the pathogenesis of allergic conjunctivitis played an irreplaceable role. However, these methods can simulate only the environmental conditions that are similar to human disease. The current study performed the human eye biopsy confirming directly the expression of TSLP and its downstream molecules in different types of patients with allergic conjunctivitis. This study provides clinical evidence for human ocular allergic diseases and provides a reference for conducting similar research work on ocular allergic diseases later. However, identifying the roles of TSLP and its downstream molecules in different types of allergic conjunctivitis needs more research.

## Figures and Tables

**Table 1:** Tear TSLP, IL-4, IL-5, and IL-13 cytokine concentrations in each group ($\overset{-}{x}\pm s$, pg/mL).

Group	Sample size	TSLP	IL-4	IL-5	IL-13
Control group	20	0.00 ± 0.00	0.00 ± 0.00	0.00 ± 0.00	0.00 ± 0.00
VKC group	20	48.91 ± 7.45	14.06 ± 3.50	10.88 ± 1.82	34.28 ± 8.42
SAC group	20	24.63 ± 2.43	7.71 ± 0.65	5.10 ± 1.33	23.77 ± 6.29
PAC group	20	21.56 ± 2.72	3.30 ± 1.50	2.43 ± 1.28	17.67 ± 4.28
*F*		349.71	200.29	260.49	128.23
*P*		<0.001	<0.001	<0.001	<0.001

TSLP, IL-4, IL-5, and IL-13 concentrations in the control group were not detected, but these were detected in VKC, SAC, and PAC patient specimens. The results of the four different types of allergic conjunctivitis are shown as mean ± SD. The Luminex microbead assay confirmed that the patient groups had different degrees of the increase in concentration of the cytokines compared with the normal control group. *P* < 0.001; each group *n* = 20.

VKC: vernal keratoconjunctivitis; SAC: seasonal allergic conjunctivitis; PAC: perennial allergic conjunctivitis.
